# A truncated nisin variant, lipidated at its C-terminus, displays improved stability and increased antibacterial activity against methicillin-resistant *Staphylococcus aureus*

**DOI:** 10.1039/d6cb00193a

**Published:** 2026-09-07

**Authors:** Longcheng Guo, Chenhui Wang, Oscar P. Kuipers, Jaap Broos

**Affiliations:** a Department of Molecular Genetics, Groningen Biomolecular Sciences and Biotechnology Institute, University of Groningen Nijenborgh 7 Groningen 9747 AG Netherlands j.broos@rug.nl; b Department of Chemistry, The University of Hong Kong Pokfulam Road Hong Kong China

## Abstract

The rise of bacterial resistance has prompted researchers to shift their focus toward alternative antimicrobial agents, including antimicrobial peptides (AMPs). Some naturally occurring AMPs are lipidated, showing similarity with clinically used antimicrobial agents such as daptomycin and polymyxins, which are lipidated peptides. In this study, we investigate the impact of various lipid modifications on nisin. Nisin is a well-studied 34-amino acid AMP, ribosomally synthesized and post-translational modified, which is effective against many Gram-positive bacteria and is widely used as a food preservative. Wild-type nisin and two truncated forms, nisin(1–31) and nisin(1–20) were conjugated at the C-terminus with either a C4, C6, C8, or C10 lipid tail. Antimicrobial activity was assessed against two Gram-positive strains: methicillin-resistant *Staphylococcus aureus* (MRSA) and vancomycin-resistant *Enterococcus faecium* (VRE). Our results show that the lipidation of wild-type nisin and nisin(1–31) reduced its antibacterial activity, with the longest chains exhibiting the lowest inhibitory effects. However, lipidation of the nisin fragment nisin(1–20), especially with C8 resulting in compound 12, enhanced its antibacterial activity and specificity against *S. aureus* species, making it four times more potent than wild-type nisin. Mode-of-action studies revealed that the lipidated construct 12 retained its ability to bind lipid II but does not induce pore formation in *S. aureus*. Notably, due to its short length, ring-protected structure, and lipid modification, the construct demonstrated improved proteolytic stability against chymotrypsin, trypsin and protease K. Our findings suggest that optimizing lipid–peptide combinations can lead to more effective, specific, and stable candidates for antimicrobial development.

## Abstract 2

Balanced lipid–peptide combinations can lead to more effective, specific, and stable candidates for antimicrobial development.

## Graphical abstract

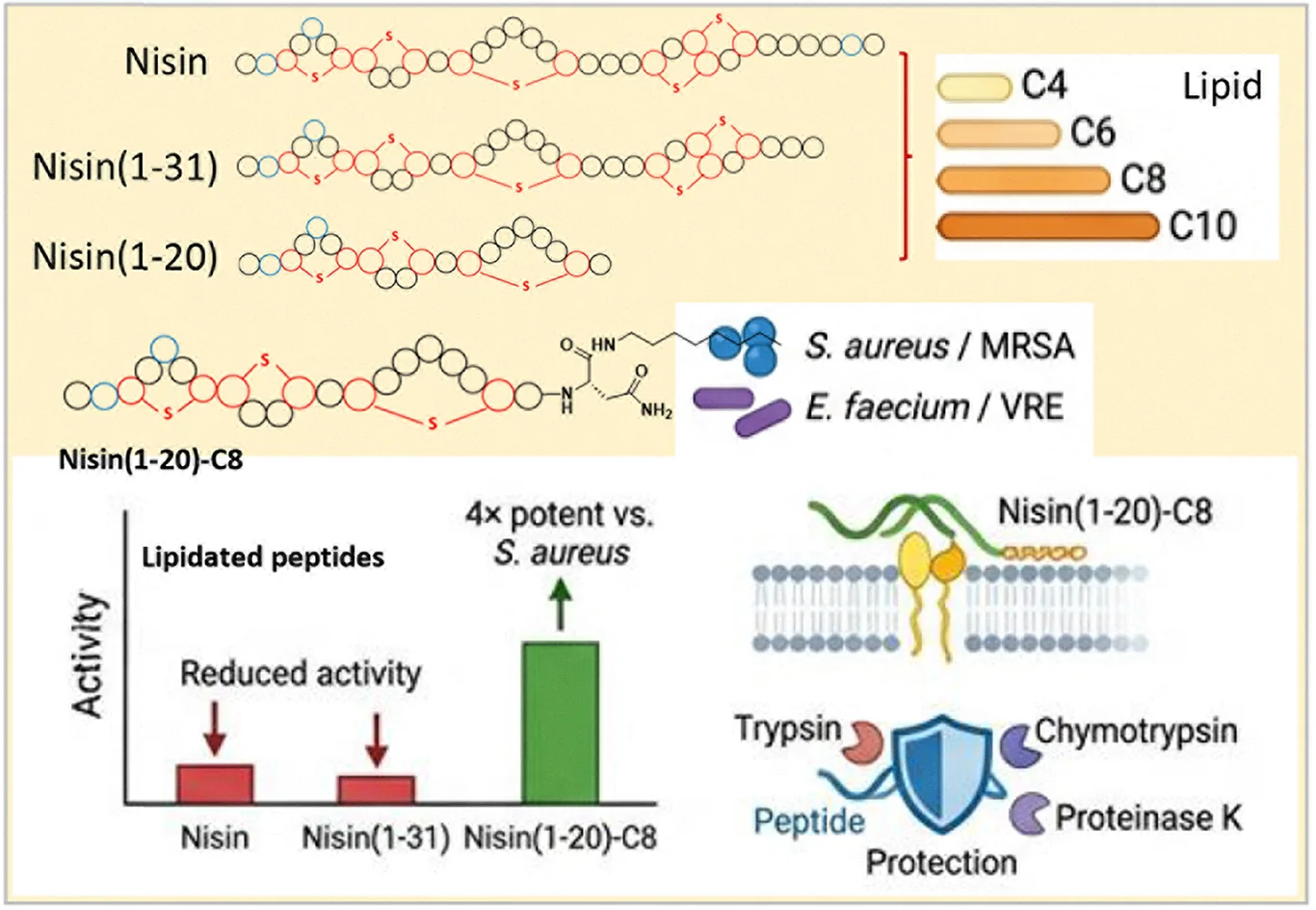

## Introduction

Since their introduction in the 1930s, antibiotics have been celebrated as one of the most significant medical breakthroughs of the 20th century.^1^ Access to effective antibiotics is crucial for society, as many medical procedures, such as organ transplants, surgeries and chemotherapy rely heavily on these antimicrobial agents to reduce the risk of infection.^2^ However, the rise of drug-resistant organisms threatens the continued effectiveness of antibiotics, as these pathogens develop resistance to one or more types of antimicrobial drugs.^3^ Today, we face a growing gap between the increasing number of antibiotic-resistant bacteria and the slow pace of discovering new antibiotics with novel mechanisms of action.^4^ If current trends persist, it is projected that by 2050, antimicrobial resistance (AMR) could cause up to 10 million deaths annually,^5^ making it one of the leading causes of death worldwide, surpassing diseases like cancer.

Peptide based antibiotics, including vital “antibiotics of last resort” like vancomycin and daptomycin^6^ and antimicrobial peptides (AMPs),^7^ have gained attention as promising agents against resistant pathogens.^8^ Unlike conventional small-molecule antibiotics that usually focus on a single bacterial target, peptide antibiotics act through a variety of unique mechanisms, primarily by disrupting cell membranes or targeting specific (intra)cellular components, allowing them to inhibit a broad spectrum of microorganisms.^9^

Currently, the clinically used peptide antibiotics are predominantly derived from the class known as non-ribosomally produced peptides (NRPs). The biosynthesis of NRPs involves NRP synthetases (NRPSs), which function in a modular, assembly-line fashion. These NRPS enzymes are large proteins, ranging from approximately 220 kDa to 2.2 MDa, characterized by high substrate specificity and encoded by extensive gene clusters. This complexity poses significant challenges for efficient engineering.^10,11^ The structures of these peptide antibiotics often feature modifications such as cyclization, unnatural amino acids, and lipidation^6^ modifications which were found essential for maintaining structure, stability and antibacterial activity.^12^ Structure-activity relationship studies of polymyxins, for example, emphasize that the fatty acyl chain is vital for their antimicrobial effectiveness.^13^ A notable study by Makovitzki *et al.* demonstrated that short peptides (4-mers), when conjugated with various lipid chains, can exhibit potent antimicrobial activity.^14^

In this study, we investigate the antimicrobial potential of wild-type nisin and truncated nisin variants after lipidation at the C terminus. Nisin is one of the best studied AMPs and belongs to the ribosomally synthesized and post-translationally modified peptide (RiPP) family.^15,16^ As a post-translationally modified peptide of 34 amino acids, nisin is stabilized by ring structures and contains unnatural amino acids (Fig. 1a), like NRP antibiotics.

**Fig. 1:**
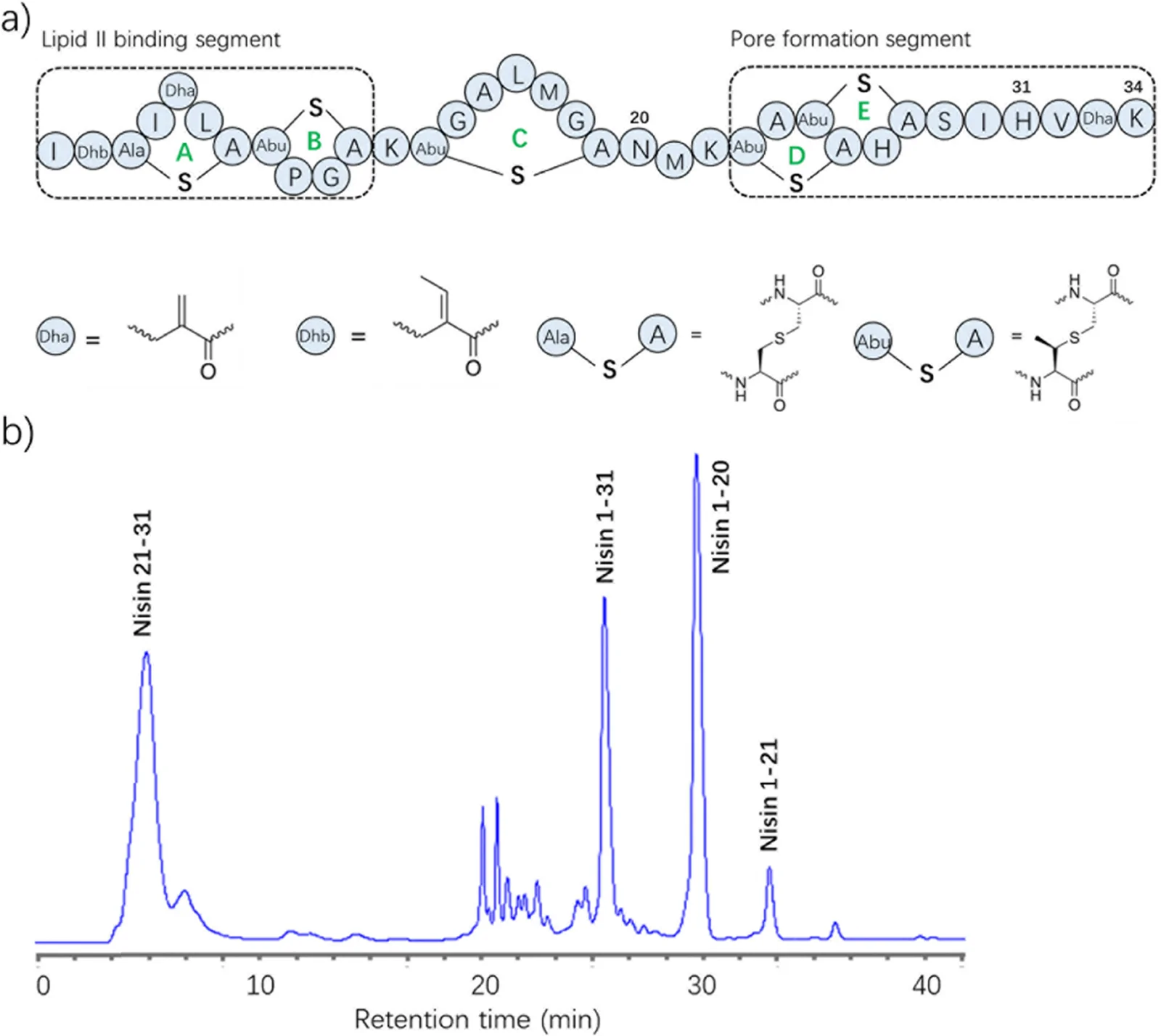
Nisin structure and its α-chymotrypsin degradation fragments. (a) Structural representation of nisin. The functional domains, encompassing the N-terminal lipid II binding site and the C-terminal pore formation domain, are indicated. Dha, dehydroalanine; Dhb, dehydrobutyrine; Ala-S-A, lanthionine; Abu-S-A, methyllanthionine. (b) Analytical HPLC traces after digestion for 64 h of nisin by chymotrypsin. The peaks are marked with the corresponding nisin fragments identified by MALDI-TOF MS.

Nisin binds lipid II and can form pores in bacterial membranes. A solid-state nuclear magnetic resonance (ssNMR) spectroscopy study by Medeiros-Silva *et al.* of the nisin-lipid II pore complex provides detailed structural insight about the location of the different nisin residues in the pore complex and its interaction with lipid II.^17^ The nisin chain protrudes through the membrane with the last 5 residues located at the membrane-water interface. The pyrophosphate group of lipid II is tightly bound by ring A–B residues (Fig. 1a) and pharmaceutical hotspots like the hinge residues and K12 line the pore lumen. Recent molecular dynamics (MD) studies offer more insight into the structure and membrane interactions of the nisin-lipid II pore complex. For the assembly of the 8 nisin and 4 lipid II molecules into a pore complex, a β sheet structure between residues 28–32 of two nisin molecules was found important.^18^ Lipid II stabilizes the pore complex and destabilizes the membrane structure near the pore complex, making it more lethal for host organisms.^19^

The ribosomal synthesis of nisin allows structural engineering using molecular biology techniques.^20,21^ Nisin exhibits potent activity against Gram-positive pathogens and has been used as a food preservative for decades, with minimal development of resistance.^16^ Here, we explore the effects of attaching a lipid chain at the C-terminus of full-length nisin to assess how lipidation influences its antibacterial activity. Due to nisin's peptidic nature, it faces certain limitations, such as susceptibility to proteolytic degradation,^22–24^ which hampers its therapeutic potential. To address this, we used enzymatic cleavage to generate more stable nisin fragments, namely nisin (1–31) and nisin (1–20) (Fig. 1b), and examined which impact the addition of a lipid chain has on antibacterial efficacy, modes of action and stability of the nisin variants. Earlier, Koopmans *et al.* reported that C-terminal lipidation of a short nisin fragment can result in an antibiotic activity similar to that of wild-type nisin.^25^ In this work we show this strategy can yield antimicrobial activities up to four-fold higher than wild-type nisin, and high lipopeptide stability towards proteases.

## Results and discussion

### Nisin's C-terminal lipidation reduces its antimicrobial activity

More than 15 natural nisin peptides have been identified^16,26^ (Fig. S1). The analysis of conserved residues demonstrated that all variants exhibit highly conserved N-terminal ring structures, a result of intramolecular cross-linking involving cysteine, serine, and threonine residues (Fig. S1). These residues are essential for the formation of the lanthionine rings, which are critical for the antimicrobial activity of nisin. In contrast, the C-terminal linear regions show significant variability in both length and amino acid composition, suggesting functional diversification among nisin peptide through modifications in their C-terminal regions. The schematic illustration in Fig. 2a depicts the lipidation process, in which a lipid amine is conjugated to the C-terminal lysine residue of nisin Z (referred to wild-type nisin) *via* amide bond formation. Notably, the lysine side chain amino group can undergo an intramolecular reaction with the carboxylate group, a dehydration reaction, leading to cyclization of the lysine side chain as shown in Fig. 2b and c. This modification produces compound 1, which, compared to wild-type nisin, exhibits a marked reduction in antibacterial activity against two pathogenic bacteria, *Enterococcus faecium* and *Staphylococcus aureus*. Specifically, a 17% and 53% decrease in the zone of inhibition area was observed in agar well diffusion assays (Fig. 2d), suggesting that the positively charged N-terminal lysine residue plays a critical role in nisin's antimicrobial efficacy.

**Fig. 2:**
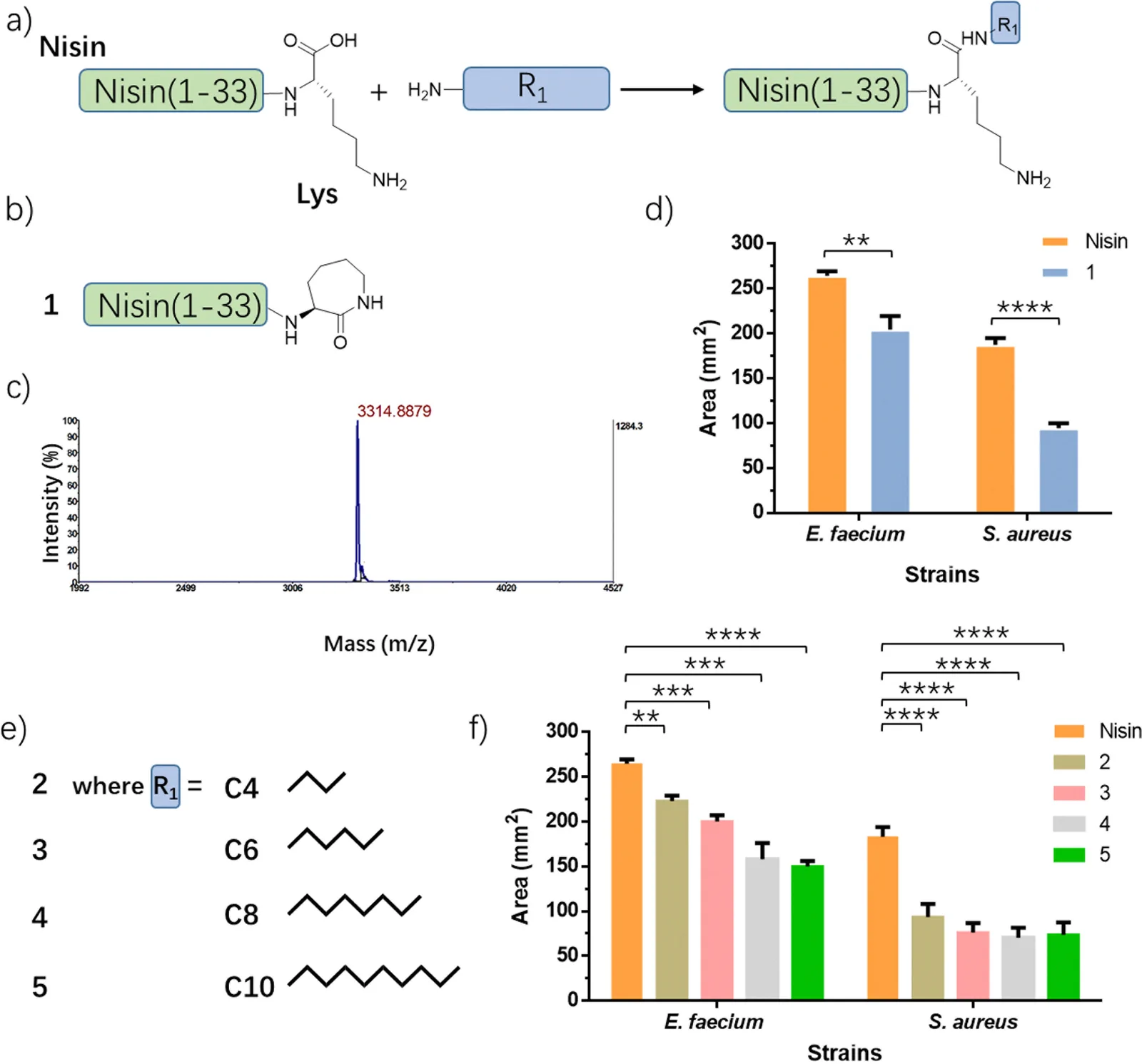
Modifications of full length nisin and their effects on antimicrobial activity. (a) Lipidation of wild-type nisin: aliphatic amine attachment to nisin is facilitated through amide bond formation of the Lys34 side chain at the C-terminal end. (b) Self-cyclization of the C-terminal Lys residue of nisin yields compound 1. (c) Detected mass of compound 1. (d) Antimicrobial activity of nisin and compound 1. Statistical analysis was performed using an unpaired two-tailed *t*-test. (e) The structure of compounds 2–5. (f) Antimicrobial activity of nisin and lipidated nisin variants: the inhibitory effects of nisin and compounds 2–5 (nisin + C4, nisin + C6, nisin + C8, and nisin + C10) were evaluated against *E. faecium* and *S. aureus*. Statistical analysis was performed using one-way ANOVA, with the unmodified compound as the control group. The bar graph quantifies the antimicrobial efficacy, measured as the inhibition zone area (mm^2^). Data is presented as mean ± SD (*n* = 3). Statistical significance was defined as ns (*P* > 0.05), * (*P* < 0.05), ** (*P* < 0.01), *** (*P* < 0.001), and **** (*P* < 0.0001).

The lipidation reaction conditions were optimized based on data presented in Fig. S2, with the highest yield of 34% achieved at a peptide to lipid-amine ratio of 400 equivalents within our tested concentration range from 20 equivalents to 400 equivalents. The antimicrobial activities of wild-type nisin and its lipidated derivatives (compounds 2, 3, 4, and 5; Fig. 2e and Fig. S4) were evaluated against *E. faecium* and *S. aureus* (Fig. 2f). Quantitative analysis of the inhibition zones revealed that the incorporation of lipid moieties significantly influences nisin's antimicrobial potency. Against *E. faecium*, wild-type nisin demonstrated the largest inhibition zone, with a trend of decreasing activity correlating with increasing lipid chain length. Variants bearing short-chain (C4 and C6; compounds 2 and 3) (Table S1) retained notable activity, whereas derivatives with longer chains (C8 and C10; compounds 4 and 5) showed further reductions in efficacy. A similar pattern was observed against *S. aureus*, where native nisin exhibited the highest antimicrobial activity, followed by nisin conjugates with C4 and C6 chains, while longer chains (C8 and C10) resulted in diminished inhibitory effects.

These findings suggest that lipidation of nisin at the C-terminus is not an optimal modification strategy,^27^ likely due to the excessive length of the construct after the last ring structure. Such modifications may interfere with the membrane translocation functions of the C-terminal region,^28^ thereby impairing antimicrobial activity.

### Lipidation of truncated nisin variants and its effect on antimicrobial activity

Koopmans *et al.* reported that attaching a lipid tail to the C-terminus of a short nisin fragment, nisin(1–12), resulted in semisynthetic lipopeptides displaying antibacterial activities on par with that of the wild-type nisin against *S. aureus* and *E. faecium*.^25^ Inspired by this work, further investigations were conducted to examine the lipidation of two truncated nisin variants: nisin(1–3l) and nisin(1–20), which are two major cleavage products when nisin is treated with chymotrypsin^23^ (Fig. 1b and Fig. S3). The schematic representations in Fig. 3a illustrate the chemical structures of the truncated peptides, highlighting the potential histidine (His) and asparagine (Asn) lipidation sites in nisin(1–31) and nisin(1–20), respectively. Lipid chains of various lengths (C4, C6, C8, and C10) were conjugated to these peptides (Fig. 3b and Fig. S5 and S6).

**Fig. 3:**
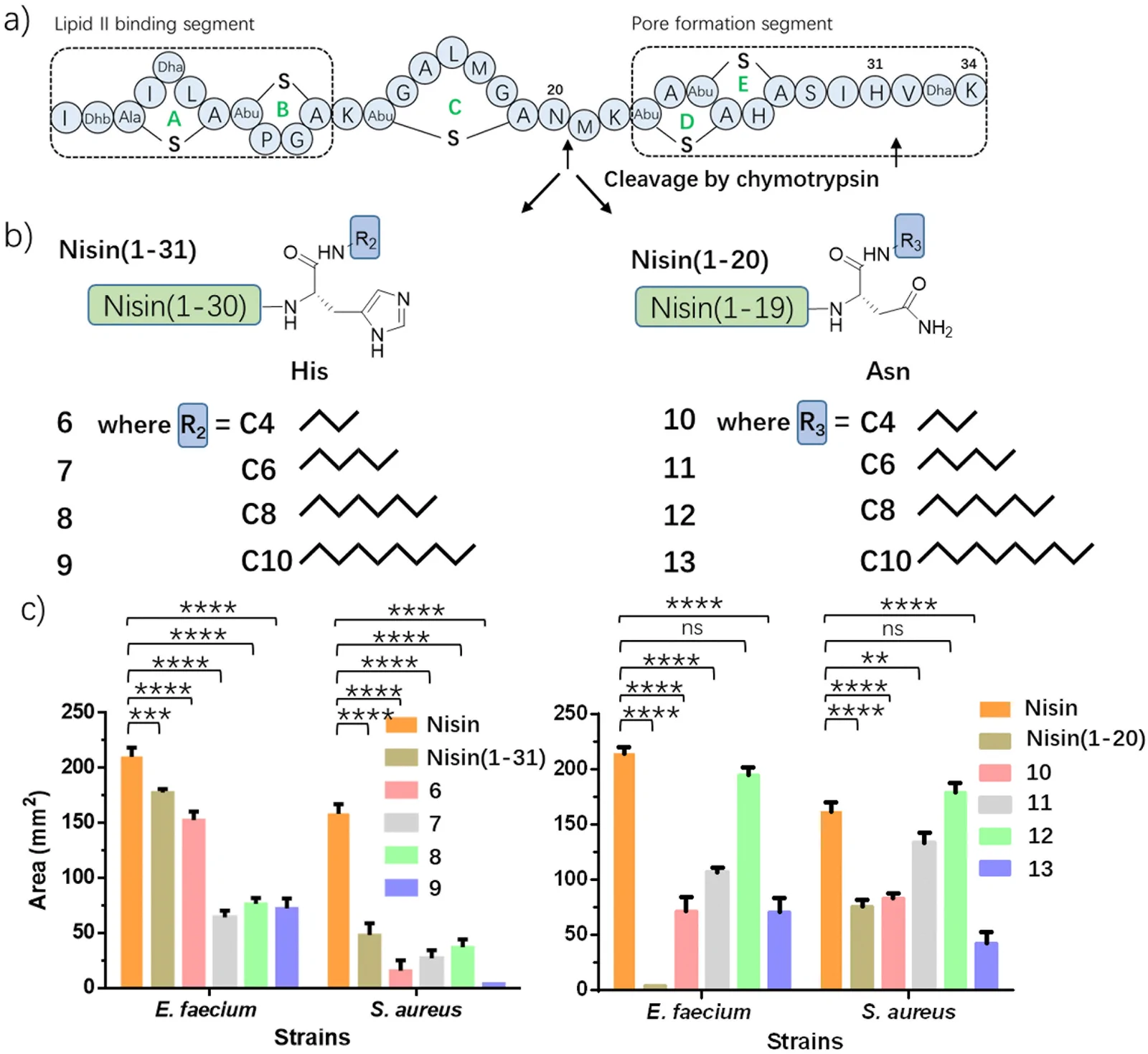
Lipidation of two truncated nisin variants, nisin(1–31) and nisin(1–20), and their antimicrobial activities against *E. faecium* and *S. aureus*. (a) Structural representation of wild-type nisin highlighting the chymotrypsin cleavage site with an arrow. (b) Nisin(1–31) and nisin(1–20) were lipidated at the histidine (His) and asparagine (Asn) residues, respectively, with various lipid chain lengths (C4, C6, C8, and C10), resulting in compounds 6–9 and 10–13. (c) Inhibition zone assays of compounds 6–13 against *E. faecium* and *S. aureus*. Statistical analysis was performed using one-way ANOVA, with the unmodified compound as the control group. The bar graph quantifies the antimicrobial efficacy, measured as the inhibition zone area (mm^2^). Data is presented as mean ± SD (*n* = 3). Statistical significance was defined as ns (*P* > 0.05), * (*P* < 0.05), ** (*P* < 0.01), *** (*P* < 0.001), and **** (*P* < 0.0001).

The antimicrobial activities of wild-type nisin, nisin(1–31), nisin(1–20) and its lipidated derivatives were evaluated against *E. faecium* and *S. aureus* (Fig. 3c). The results indicate that wild-type nisin exhibited the highest antimicrobial activity against *E. faecium* followed by nisin(1–31), which retained moderate activity. However, lipidation of nisin(1–31) at the C-terminus resulted in a decline in antimicrobial activity, with introducing the longest lipid chains (C6, C8 and C10) yielding the lowest inhibitory effects. Against *S. aureus*, nisin(1–3l) and its lipidated derivatives demonstrated limited or negligible antimicrobial activity. These findings suggest that lipidation at this position may not be a suitable modification for enhancing bioactivity.

Conversely, a different trend was observed for nisin(1–20) and its lipidated variants (Fig. 3c). While nisin(1–20) exhibited essentially no antimicrobial activity against *E. faecium*, the lipidated derivatives displayed activity. Notably, nisin(1–20) + C8 (compound 12) exhibited activities against both *E. faecium* and *S. aureus* (Fig. 3c) comparable or higher than that of wild-type nisin. Taken together, introducing a lipid chain at nisin or nisin (1–31) reduces its activity against both tested pathogens. The longer the lipid chain, the lower the activity of the lipopeptide. This suggests the introduced lipid tail interferes with nisin pore assembly, a process where the peptide chain proceeds through the membrane, with the last C-terminal residues positioned at the membrane water interface.^17^ In contrast, our findings indicate that attaching the lipid tail to nisin(1–20) enhances activity, with the highest efficacy observed when using a C8 tail. Koopmans *et al.* introduced a linear C6, C10 or C14 alkyl chain at the C-terminus of nisin (1–12) and found highest activity for the C14 construct while the C6 construct showed minimal activity.^25^ Thus, a more truncated nisin (nisin(1–12) *versus* nisin(1–20)) benefits from a longer C-terminal alkyl tail (C14 *versus* C8) resulting in activities of these constructs being comparable or better than nisin. Other lipopeptide engineering studies have also found a strong dependence of antimicrobial activity on tail and peptide structure.^12^ The chain length of the fatty acid is critical; both too short and too long chains can diminish efficacy, possibly due to inadequate membrane anchoring or issues like toxicity, self-aggregation, or limited solubility. Complex structure activity relationships were observed, indicating that not all steps in the antibacterial action of lipopeptides are well understood. One trend observed in these studies is the longer the lipid tail, the more toxic the lipopeptide becomes for human erythrocytes, limiting its pharmaceutical application.^12^

### Compound 12 shows improved antibacterial activity against *S. aureus* species

To further investigate how lipidation influences the antimicrobial activity of truncated nisin variant nisin(1–20), the MIC values of wild-type nisin, nisin(1–20) and compound 12 (Fig. S7) were assessed against four pathogenic Gram-positive bacteria: *Staphylococcus aureus*, *Enterococcus faecium*, *Bacillus cereus*, and *Listeria monocytogenes* (Table 1). The results showed that wild-type nisin had strong activity, with MICs of 0.5 µg mL^−1^ for *E. faecium*, 7.6 µg mL^−1^ for *S. aureus*, and 15.2 µg mL^−1^ against both *B. cereus* and *L. monocytogenes*. In contrast, nisin(1–20) alone exhibited a significant reduction in potency, with an MIC of 30.4 µg mL^−1^ against *S. aureus* and exceeding 121.5 µg mL^−1^ for the other three pathogens, indicating a substantial loss of activity following truncation.^29,30^

**Table 1:** Antimicrobial profile of wild-type nisin, nisin(1–20) and compound 12 against selected Gram-positive strains^a^

Organism and type	MIC (µg mL^−1^)		
	Nisin	Nisin(1–20)	12
*Staphylococcus aureus* LMG15975 (MRSA)	7.6	30.4	1.9
*Listeria monocytogenes* LMG10470	15.2	>121.5	30.4
*Enterococcus faecium* LMG11423	0.5	121.5	3.8
*Bacillus cereus* CH-85	15.2	121.5	7.6

a MRSA, methicillin resistant *Staphylococcus aureus*.

However, lipidation of nisin(1–20) with an octyl (C8) group, resulting in compound 12, markedly improved antimicrobial efficacy. Against *S. aureus*, the MIC value decreased to 1.9 µg mL^−1^, making it 16 times more effective than unmodified nisin(1–20) and, interestingly, four times more potent than wild-type nisin. Additionally, activity against the other three pathogens was restored, with efficacy up to 32 times higher than unmodified nisin(1–20). Compared to wild-type nisin, compound 12 showed a two-fold increase in activity against *B. cereus*, but a two-fold and eight-fold decrease against *L. monocytogenes* and *E. faecium*, respectively, suggesting that lipidation alters the antimicrobial spectrum of the compound. Further analysis using a panel of strains, including two *S. aureus* strains, two *E. faecium* strains, one *E. faecalis*, one *L. monocytogenes*, one *B. cereus*, and one Gram-negative *Escherichia coli* strain (Table S2), revealed that compound 12 was highly potent against *S. aureus*, *B. cereus*, and *E. faecalis*. Conversely, wild-type nisin remained more effective against *E. faecium* and *L. monocytogenes* (Table 2 and Fig. S8). Neither peptide showed activity against *E. coli*.

**Table 2:** Antimicrobial activity of wild-type nisin, nisin(1–20) and compound 12 against pathogenic microorganisms^b^

Organism and type^a^	Nisin	Nisin(1–20)	12
*Staphylococcus aureus* LMG10147	++	+	++++
*Staphylococcus aureus* LMG15975 (MRSA)	++	+	++++
*Bacillus cereus* CH-85	++	+	+++
*Enterococcus faecalis* LMG16216 (VRE)	++	+	++++
*Enterococcus faecium* LMG16003 (VRE)	++++	—	+++
*Enterococcus faecium* LMG11423	+++++	—	+++
*Listeria monocytogenes* LMG10470	++	—	+
*Escherichia coli* CECT101	—	—	—

a VRE, vancomycin-resistant enterococci; MRSA, methicillin resistant *Staphylococcus aureus*.

b Note: the diameter (mm) was determined using the spot-on-lawn assay (Fig. S8) in triplicate, with corresponding ratings as follow: 0–4 mm, +; 4–7 mm, ++; 7–9 mm, +++; 9–12 mm, ++++; and 12–15 mm, +++++.

Together, this MIC study shows compound 12 is 16-fold more active than nisin(1–20) against the tested *S. aureus* and *B. cereus* strains and 4 and 2 times, respectively, more active than wild-type nisin against these two strains.

### Compound 12 binds lipid II without inducing pore formation

Nisin employs a unique dual mode of antimicrobial action: lipid II trafficking and pore formation.^18,31–34^ The lipid II interaction is mediated by the lipid-binding domain formed by rings A and B^18,31^ (Fig. 1a). Notably, the truncated peptide nisin(1–20) retains this lipid-binding domain (Fig. 3b). Consistent with its structure, compound 12 was able to bind lipid II, as evidenced by decreased antimicrobial activity against *S. aureus* when lipid II was added near the antibiotics, indicating competitive binding (Fig. 4a). A similar pattern was observed with full-length nisin, but not with daptomycin,^35^ which does not target lipid II.

**Fig. 4:**
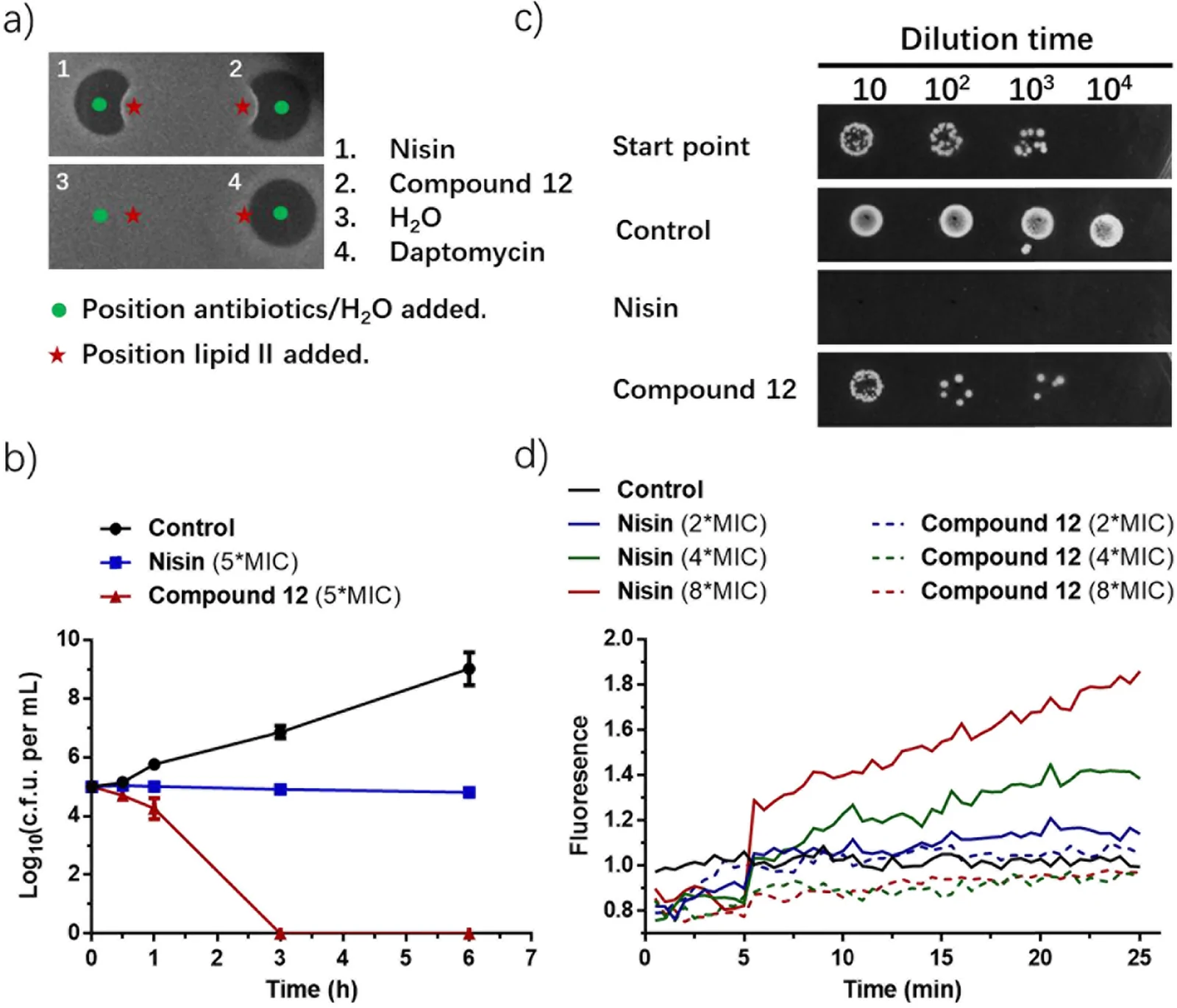
Lipid II binding, bactericidal activity, and pore-forming ability of nisin compound 12. (a) A spot-on-lawn assay assessed binding to the cell wall precursor lipid II. Nisin served as a positive control; daptomycin and H_2_O were negative controls. (b) Time-kill curves evaluated the bactericidal/bacteriostatic activity of nisin and compound 12 against *S. aureus*, with H_2_O as a control. (c) *S. aureus* cells treated with antibiotics at 5× MIC for 6 hours were examined; solutions were spotted at indicated dilution factors, with 5 µL applied. The initial concentration is marked as the starting point. (d) Potassium leakage assay measured pore formation by detecting fluorescence increase of the PBFI probe after adding various antimicrobial concentrations. Antibiotics were added at 5 minutes; H_2_O served as a control.

In addition to inhibiting cell wall synthesis, nisin kills bacteria by inducing membrane pore formation.^36^ This activity involves the hinge region, rings D and E, and part of the tail region^17^ (Fig. 1a). However, these parts are absent in nisin(1–20) and compound 12. Previous studies have shown that nisin(1–22) has a bacteriostatic effect, as it can bind lipid II and halt cell growth but does not cause cell death.^37^ To further explore the mechanisms underlying the enhanced antimicrobial activity of lipidated nisin(1–20), we conducted a time-kill assay and a membrane pore formation assay (Fig. 4). The time-kill experiment monitored bacterial viability over time following treatment with nisin or compound 12. Results showed that untreated bacteria continued to grow, while compound 12 inhibited bacterial proliferation without significantly reducing colony-forming units (CFUs) (Fig. 4b and c), indication compound 12 shows no bactericidal activity. In contrast, full-length nisin exhibited rapid bactericidal activity, completely eradicating bacteria within three hours (Fig. 4b).

The pore formation assay aimed to assess the peptides ability to permeabilize bacterial membranes (Fig. 4d). Using a fluorescence-based method, we observed that nisin caused a concentration-dependent increase in fluorescence, indicating moderate membrane permeabilization. Conversely, compound 12 showed significantly lower fluorescence increases, even at concentrations eight times the MIC, suggesting no pore-forming activity.^30^ These findings indicate that lipidation markedly improves the antimicrobial activity of nisin(1–20) without promoting membrane permeabilization. The antimicrobial activity of compound 12 is therefore likely only due to lipid II trafficking and introduction of a C8 tail at nisin(1–20) may facilitate proper nisin-lipid II complex assembly bringing the N-terminal lipid II binding domain in a good position to bind lipid II. Interestingly, the experiments with *S. aureus* cells in Fig. 4 show nisin binds lipid II and induce cell leakage, yet its MIC value is 4 times higher than for compound 12, not showing the latter activity. One explanation for this could be a higher stability of compound 12 than nisin in the MIC test. Below the stability of both compounds is addressed.

### Compound 12 demonstrates enhanced stability against proteolytic degradation

A significant challenge for the therapeutic application of nisin is its susceptibility to proteolytic cleavage by intestinal enzymes.^38^ Nisin has been shown to be vulnerable to proteases such as trypsin and chymotrypsin, with cleavage sites identified at positions including Lys12, Asn20, Met21, Lys22, and His31.^39^ To address this issue, we hypothesized that the synthetic variant, compound 12, which lacks most of these protease cleavage sites, would exhibit enhanced resistance to proteolytic degradation (Fig. 5a).

**Fig. 5:**
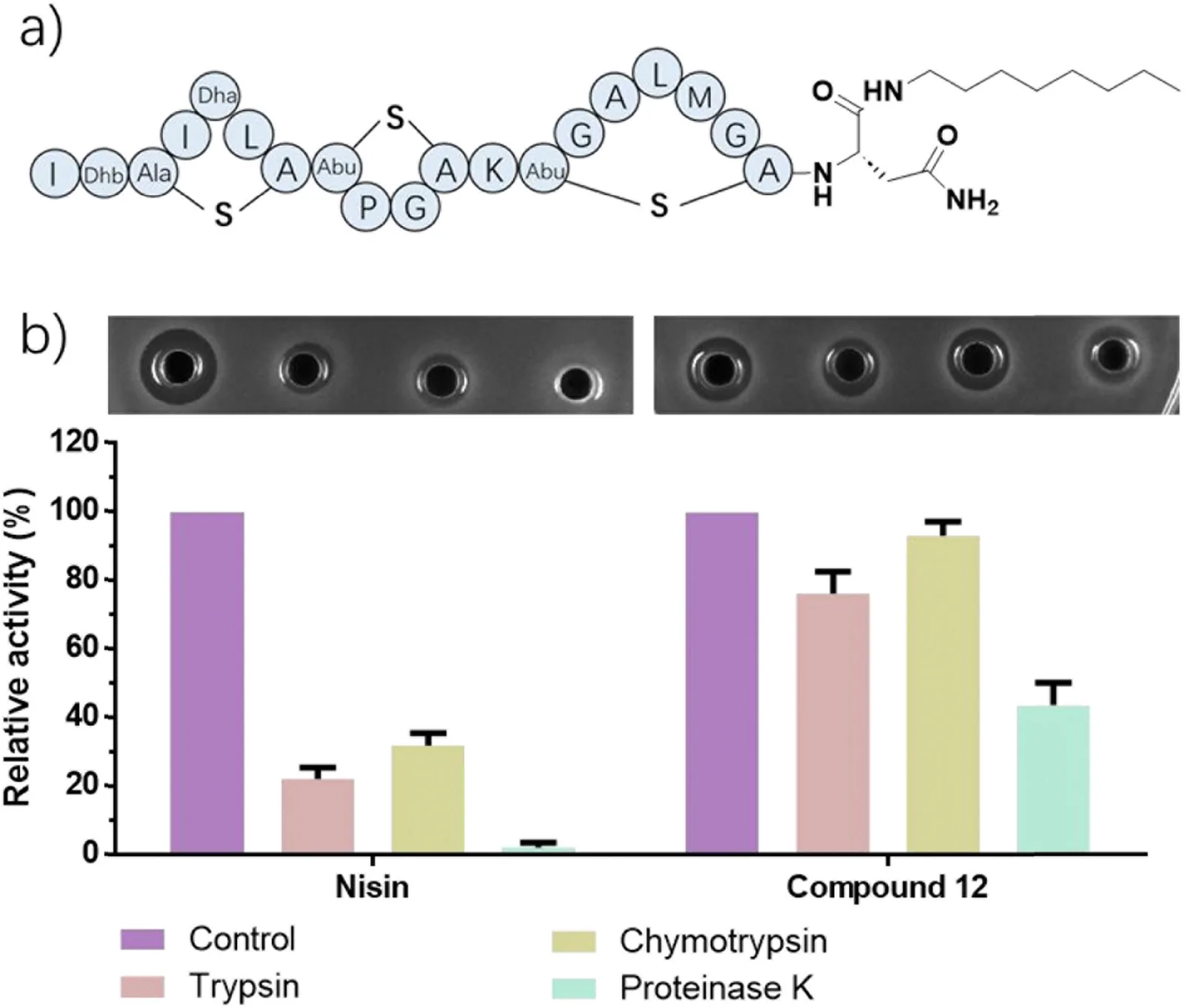
Susceptibility of nisin and nisin(1–20) + C8 (compound 12) when exposed to different proteolytic enzymes. (a) Structural representation of compound 12, highlighting the lipidation site at the C-terminus. (b) Relative antimicrobial activity toward *L. lactis* MG1363 of nisin and compound 12 without (control) or with exposure to various proteolytic enzymes. A representative image from three independent experiments was presented.

To test this, we evaluated the overnight susceptibility of both nisin and compound 12 to inactivation by trypsin, chymotrypsin, and proteinase K using an agar diffusion assay (Fig. 5b). The results showed that wild-type nisin experienced varying degrees of stability: approximately 78% activity loss with trypsin, 70% with chymotrypsin, and near-complete activity loss when exposed to proteinase K. Conversely, compound 12 demonstrated markedly improved resistance to these proteases, as less than 25% activity was lost when exposed to trypsin or chymotrypsin. Even during overnight exposure to proteinase K 42% activity remained. The high stability of compound 12 toward trypsin is remarkable as a trypsin cleavage site is present at Lys12. This suggests the C8 tail limits trypsin access to Lys12. We note that trypsin cleavage of nisin can yield in part also nisin(1–20) and nisin(1–34) fragments which escaped cleavage at Lys12.^23^

In summary, the increased resistance to enzymatic degradation of compound 12, compared to nisin, likely significantly boosted its activity, making it more active than nisin against *S. aureus* and *B. cereus*. This stability enhancement is important because the limited stability of nisin hinders its development as a therapeutic drug.^40^

## Experimental

### Materials and bacterial strains

All chemicals, unless otherwise specified, were purchased from Sigma-Aldrich (St. Louis, MO) and used directly without additional purification. Nisin Z was obtained from Handary (Brussels, Belgium) under the brand name NisinZ® P (Ultrapure Nisin Z). Detailed information regarding the aliphatic amines employed in this study is provided in Table S1. The bacterial strains utilized, and their growth conditions are summarized in Table S2.

### Nisin fragment preparation

Nisin fragment preparation was carried out following a previously described protocol.^23^ Briefly, nisin Z (60 mg, Ultrapure (>95%), Handary, Brussels, Belgium) was dissolved in 50 mL of TRIS-acetate buffer (25 mM, pH 7.5) and kept on ice. α-Chymotrypsin (5 mg) was then added to the solution, and the mixture was allowed to warm to room temperature. The enzymatic digestion proceeded at 30 °C. After 16 hours, an additional 5 mg of chymotrypsin was added, and the mixture was incubated for another 24 hours at 30 °C. This addition and incubation cycle was repeated once more. After a total of 64 hours, the reaction was acidified to pH 4 using aqueous 1 N HCl. The resulting solution was then freeze-dried. The lyophilized sample was dissolved in 0.05% acetic acid solution and subjected to further purification *via* HPLC using an Agilent 1200 series system equipped with a C_12_ column (Jupiter 4 µm Proteo 90Å, 250 × 4.6 mm, Phenomenex). The target peak was collected, lyophilized, and stored at 4 °C until further analysis.

### Nisin-lipid conjugation optimization

Since the last amino acid of nisin, lysine, contains a free amine group capable of undergoing an intramolecular reaction with its carboxyl group during the lipidation reaction, we investigated the optimal conditions for nisin to react with aliphatic amines. Specifically, different coupling reagents were tested, including BOP (benzotriazole-1-yl-oxy-tris(dimethylamino)phosphonium hexafluorophosphate) and PyBOP (Benzotriazole-1-yl-oxy-tris-pyrrolidino-phosphonium hexafluorophosphate). Nisin was dissolved in DMF (100 µL), and then butylamine (50 equivalents), along with either BOP or PyBOP (2 equivalents), and DiPEA (4 equivalents), were added. The reaction mixture was stirred at room temperature for 1 hour, then quenched with 2 mL of buffer A (H_2_O : MeCN, 95 : 5 + 0.1% TFA). The mixture was centrifuged at 5000 rpm for 5 minutes to remove insoluble material, and the supernatant was analyzed *via* HPLC.

The effect of reaction time on nisin coupling efficiency was studied by varying the duration from 10 minutes to 6 hours, using the same reaction conditions: nisin dissolved in 100 µL of DMF, along with butylamine (50 equivalents), BOP (2 equivalents), and DiPEA (4 equivalents). Additionally, the impact of different butylamine concentrations (ranging from 20 to 400 equivalents) was assessed by adding BOP (2 equivalents) and DiPEA (4 equivalents), and the mixture was stirred for 6 hours at room temperature.

### Amide coupled lipid-nisin fragment

The nisin fragment was dissolved in 240 µL of DMF. Then, the corresponding lipid-amine (50 equivalents), BOP (2 equivalents), and DiPEA (4 equivalents) were added. The reaction mixture was stirred for 6 hours and then quenched with 4 mL of buffer A (H_2_O : MeCN, 95 : 5 + 0.1% TFA). The mixture was centrifuged at 5000 rpm for 5 minutes to remove any insoluble material. The supernatant was then purified using HPLC, and the fractions containing the product were lyophilized to obtain the final lipid-nisin conjugates.

### Mass spectrometry analysis

A volume of 1 µL of the compound was spotted onto the target plate, dried, and rinsed several times with Milli-Q water. Then, an equal volume of matrix solution, comprising 5 mg mL^−1^ α-cyano-4-hydroxycinnamic acid dissolved in 50% acetonitrile with 0.1% trifluoroacetic acid, was applied on top of the sample. Mass spectra were acquired using an Applied Biosystems 4800 Plus MALDI-TOF mass spectrometer, operating in linear mode with external calibration.

For structural analysis of compound 12, high-resolution liquid chromatography–tandem mass spectrometry (HRLC-MS/MS) was performed. The analysis used a Shimadzu LC20 XR-series HPLC system coupled to a Q-Exactive mass spectrometer, equipped with an Agilent Pursuit XRs C_8_ column (50 × 2 mm). A 1 µL sample was injected and separated using a 2–95% gradient of acetonitrile containing 0.1% formic acid at a flow rate of 0.3 mL min^−1^ over 10 min. MS/MS data were collected in PRM mode, focusing on the doubly or triply charged ions of the target compound.

### Agar well diffusion assay

An overnight culture was diluted to 0.1% (v/v) and added to molten GM17 agar (for *E. faecium* LMG16003) or LB agar (for *S. aureus* LMG15975) at 45 °C. About 30 mL of this mixture was poured onto a plate and allowed to solidify. Once the agar was set, 8 mm wells were punched into the agar and filled with 30 µL of a 1 mg mL^−1^ antibiotic solution. The plates were then incubated at 37 °C overnight. After incubation, the zones of inhibition around each well were measured. The zone diameters were recorded in millimeters and expressed as the area of the zone (π*r*^2^) minus the area of the well (π*r*^2^), also in millimeters. The results shown are from three independent experiments.

### Minimal inhibitory concentration assay

The minimum inhibitory concentration (MIC) was determined using the broth microdilution method in accordance with standard protocols.^41^ The bacterial inoculum was prepared to approximately 5 × 10^5^ CFU mL^−1^. The MIC was defined as the lowest concentration of the antimicrobial agent that prevented visible bacterial growth after overnight incubation at 37 °C. All experiments were performed in triplicate.

### Effects of proteolytic enzymes on the antibacterial activity of nisin and compound 12

The effect of proteolytic enzymes on the antimicrobial activity of compound 12 was evaluated using *Lactococcus lactis* MG1363 strain *via* an agar well diffusion assay.^42^ Thirty microliters of the HPLC-purified nisin or derivative (1 mg mL^−1^) were directly added into a well on the agar plate at pH 7. This was done either in the presence of 1 mg mL^−1^ of proteolytic enzyme (final concentration) or without enzyme (serving as the control). The plates were incubated overnight at 37 °C, and the zones of inhibition were subsequently measured.

### Spot-on-lawn assay to measure peptide–lipid II complex formation

To investigate the interaction between the peptide and lipid II,^43,44^ an overnight culture of *S. aureus* was added to 0.8% (w/v) LB agar at 45 °C containing a final concentration of 0.1% (v/v), and this mixture was poured into 10 mL plates. The binding of the peptide to lipid II was further assessed by spotting 2 µL of purified lipid II (300 µM, a gift from Dr Eefjan Breukink, Utrecht University) at the periphery of the antimicrobial inhibition zone. Briefly, the antimicrobial agent was applied onto the agar plate, and after the drops dried, lipid II was spotted at the edge of the inhibition zone. The plates were then incubated overnight at 37 °C.

### Time-kill assay

The antimicrobial activity of nisin, and compound 12 was evaluated using a method previously described by Guo *et al*.^45,46^ Briefly, an overnight culture of *S. aureus* LMG15975 was diluted 50-fold in LB medium and incubated at 37 °C until reaching an OD_600_ of 0.5. The bacterial suspension was then adjusted to a concentration of 5 × 10^5^ CFU mL^−1^. The bacteria were subsequently exposed to a concentration equivalent to five times the minimum inhibitory concentration (MIC) of each peptide. An untreated bacterial suspension served as a control. At designated time points, 50 µL samples were collected, and both undiluted and 10-fold serially diluted suspensions were plated onto LB agar plates. The plates were incubated overnight at 37 °C, after which colonies were counted and expressed as CFU mL^−1^. All experiments were performed in triplicate.

### Potassium ion efflux assays

For the K^+^ release assay, the K^+^-sensitive fluorescent probe PBFI was employed.^42^*S. aureus* was cultured in LB medium until reaching an OD_600_ of 0.6. The cells were then harvested by centrifugation at 5000g for 5 minutes and washed twice with 10 mM HEPES buffer (pH 7.2) containing 0.5% glucose. The washed cells were resuspended in the same buffer supplemented with 10 µM PBFI. Fluorescence measurements were performed using a Varioskan LUX multimode microplate reader (Thermo Fisher Scientific), with excitation at 346 nm and emission recorded at 505 nm to establish a baseline signal prior to adding various concentrations of antibiotics. Nisin served as the positive control in this experiment.

## Conclusions

This study investigated lipidation engineering strategies targeting the C-terminal modification of nisin or nisin fragments. Although most clinically used NRP antimicrobial peptides are lipidated, the outcomes of such modifications on new peptide sequences and their biological activities are often complex and unpredictable. Our data indicates that the effects are highly dependent on the peptide sequence, the lipid tail used and on the target bacterium. Direct lipidation of the C-terminus of nisin and nisin(1–31) resulted in decreased antibacterial efficacy. In contrast, lipidation of the truncated nisin(1–20) variant with an C8 chain (compound 12) significantly boosted its activity against *S. aureus*, including clinically relevant MRSA strains, making it up to four times more effective than wild-type nisin. Due to its shorter length, ring-protected structure, and lipid tail modification, the construct also exhibited significantly enhanced stability when exposed to trypsin, chymotrypsin or proteinase K. Our data indicates the improved stability of compound 12 significantly contributed to the high activity of this lipopeptide. Overall, this work demonstrates that lipidation of a lanthipeptide can introduce enhanced antimicrobial activity and improved proteolytic stability.

## Author contributions

J. B., O. P. K., and L. G. conceived the project. C. W. and L. G. carried out the experiments, analyzed data, and wrote the initial manuscript. J. B. and O. P. K. corrected the manuscript. All authors checked the final version of the manuscript and agreed on it.

## Conflicts of interest

The authors declare no competing financial interest.

## Supplementary Material

CB-007-D6CB00193A-s001

## Data Availability

The data supporting this article have been included as part of the supplementary information (SI). The SI includes additional sequence analysis of natural nisin variants, optimization of nisin lipidation conditions, mass spectrometric characterisation of peptide products and derivatives, evaluation of the antibacterial activity of compound **12**, and detailed reference data on materials and bacterial strains. See DOI: https://doi.org/10.1039/d6cb00193a.
