# Supplementary material for: A truncated nisin variant, lipidated at its C-terminus, displays improved stability and increased antibacterial activity against methicillin-resistant Staphylococcus aureus
Source: RSC Chem Biol. 2026 Sep 7. Online ahead of print. doi: 10.1039/d6cb00193a (PMC13596117; doi:10.1039/d6cb00193a)
Supplement: Supplementary file 1 [file CB-OLF-D6CB00193A-s001.pdf]

Supplementary Information (SI) for RSC Chemical Biology.
This journal is © The Royal Society of Chemistry 2026

# Supporting Information for:

## A truncated nisin variant, lipidated at its C-terminus, displays improved stability and increased antibacterial activity against methicillin-resistant *Staphylococcus aureus*

Longcheng Guo[a,b,*], Chenhui Wang[a,*], Oscar P. Kuipers[a], Jaap Broos[a,#]

[a] Department of Molecular Genetics, Groningen Biomolecular Sciences and Biotechnology Institute, University of Groningen, Groningen, 9747 AG, The Netherlands

[b] Present address: Department of Chemistry, The University of Hong Kong, Pokfulam Road, Hong Kong, China

[*] These authors contributed equally to this work.

[#] Correspondence to Jaap Broos, j.broos@rug.nl

## Table of Contents


Supplementary Figure 1. Neighbor-joining tree and multiple sequence alignment of nisin variants. S3

Supplementary Figure 2. Optimization of nisin lipidation conditions. S4

Supplementary Figure 3. MALDI-TOF MS analysis of nisin fragments obtained after digestion with α-chymotrypsin. S5

Supplementary Figure 4. MALDI-TOF MS analysis of wild-type nisin with lipid chains attached. S6

Supplementary Figure 5. MALDI-TOF MS analysis of nisin(1-31) with lipid chains attached. S7

Supplementary Figure 6. MALDI-TOF MS analysis of nisin(1-20) with lipid chains attached. S8

Supplementary Figure 7. HRLC-MS/MS analysis of HPLC-purified compound **12**. S10

Supplementary Figure 8. Spot-on-lawn assay used to evaluate the antibacterial spectrum of nisin, nisin(1-20), and compound **12**. S11

Supplementary Table 1. Detailed information about the aliphatic amines used in this study. S12

Supplementary Table 2. Bacterial strains used in this study. S13

Supplementary Table 3. Comparison of theoretical average masses and MALDI-TOF measured masses of peptides produced in this study. S14

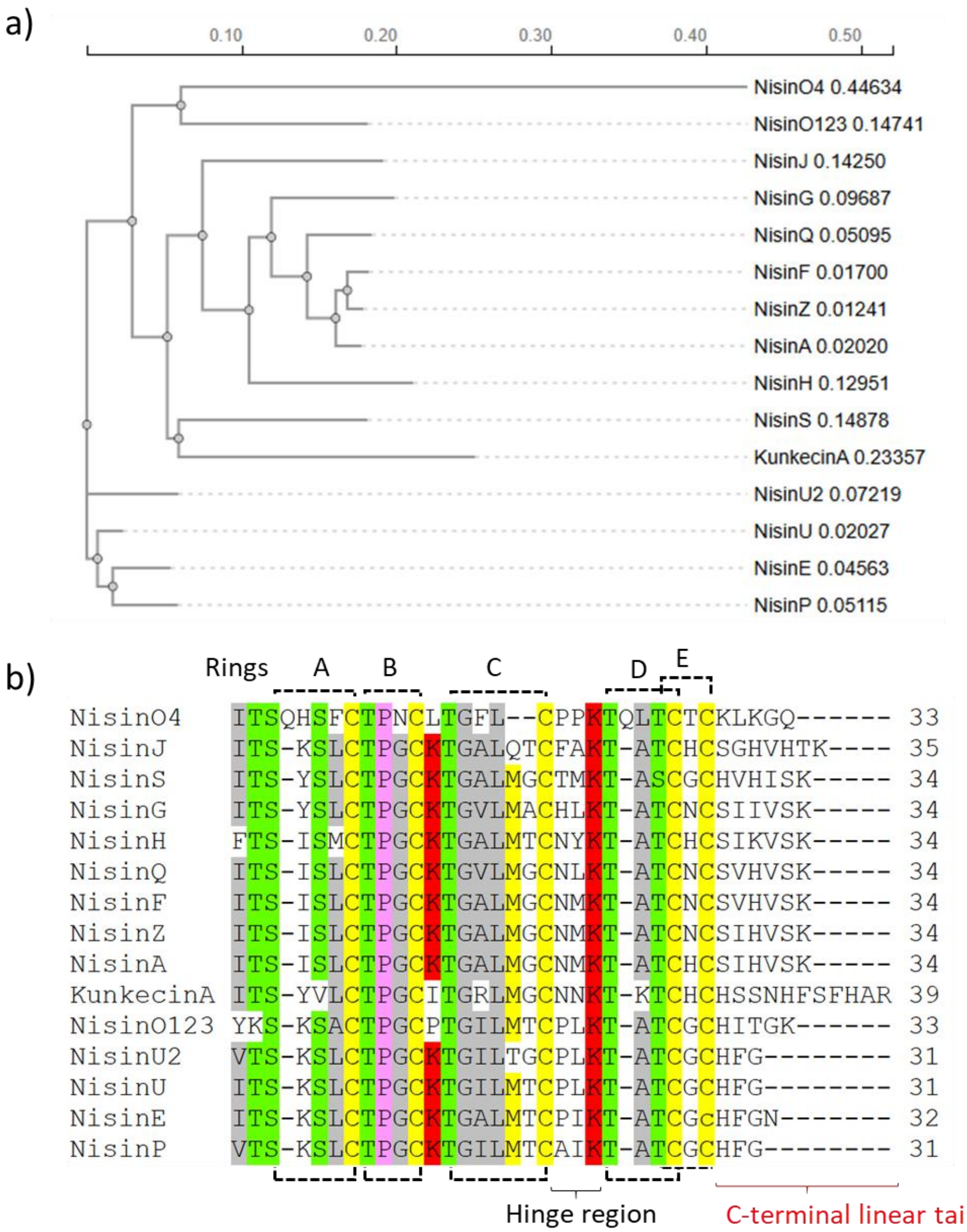


**Fig. S1** Neighbor-joining tree and multiple sequence alignment of nisin variants. (a) The dendrogram illustrates the phylogenetic relationships among natural nisin variants based on their primary structures, suggesting potential evolutionary links between the nisin variants. The branching order indicates relatedness, while branch length reflects phylogenetic distance. (b) The multiple sequence alignment of natural nisin variants was generated using MUSCLE and visualized with the tool available at https://www.bioinformatics.org/sms2/color_align_prop.html. The positions of the five (methyl)lanthionine rings are marked at the top. The numbers indicate the amino acid sequence length of each nisin variant. The variable regions, including the hinge region and the C-terminal linear tail, are highlighted.

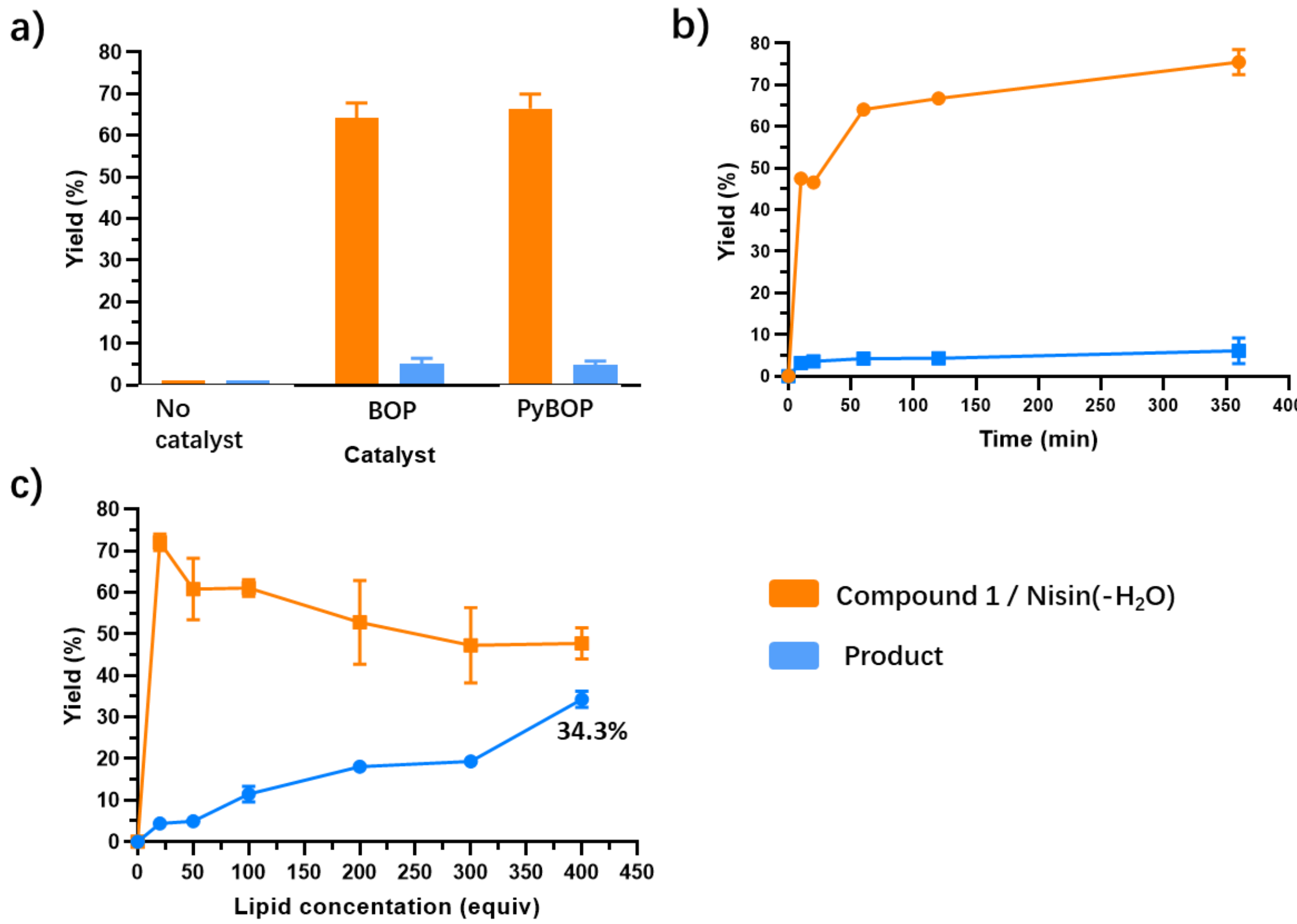


**Fig. S2** Optimization of nisin lipidation conditions. (a) Coupling Reagents Selection: Both BOP and PyBOP facilitated lipidation, producing yields of 5.2% and 4.9%, respectively, when using 50 equivalents of lipid-amine. No reaction was observed in the absence of a coupling reagent. (b) Reaction Time: The byproduct (-$H_2O$) formed rapidly within the first 50 minutes and then plateaued, while lipidation progressed more slowly, indicating a limited conversion rate over time. (c) Lipid concentration: Increasing the lipid amine to nisin ratio enhanced product formation, reaching a maximum yield of 34.3% at 400 equivalents.

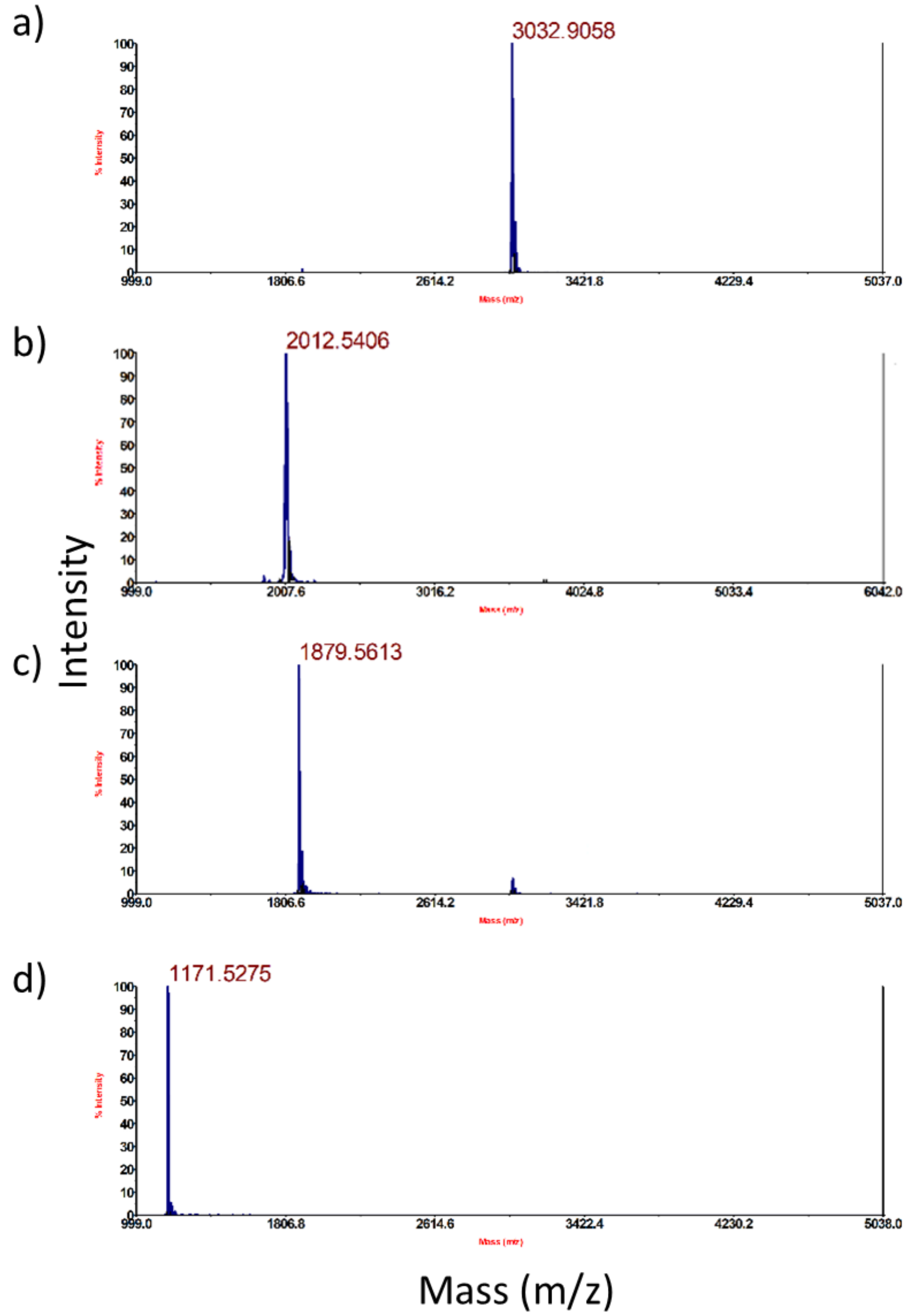


**Fig. S3** MALDI-TOF MS analysis of nisin fragments obtained after digestion with α-chymotrypsin, with the major peaks purified by HPLC. (a) MS analysis of nisin(1–31). (b) MS analysis of nisin(1–21). (c) MS analysis of nisin(1–20). (d) MS analysis of nisin(21–31). The predicted and observed masses are provided in Table S3.

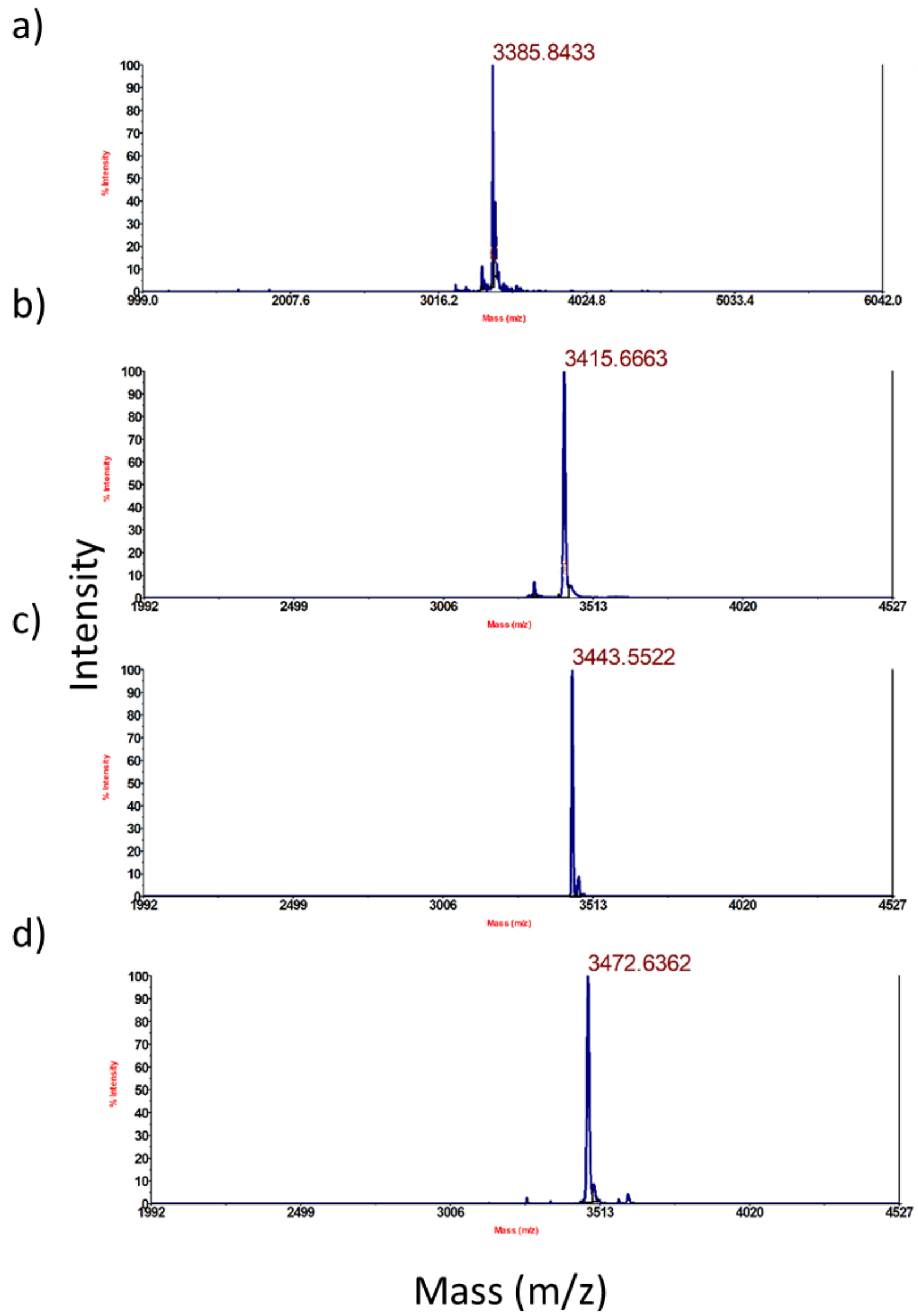


**Fig. S4** MALDI-TOF MS analysis of full length nisin with different lipid chains attached. (a) MS analysis of nisin+C4 (compound **2**). (b) MS analysis of nisin+C6 (compound **3**). (c) MS analysis of nisin+C8 (compound **4**). (d) MS analysis of nisin+C10 (compound **5**). The predicted and observed masses are provided in Table S3.

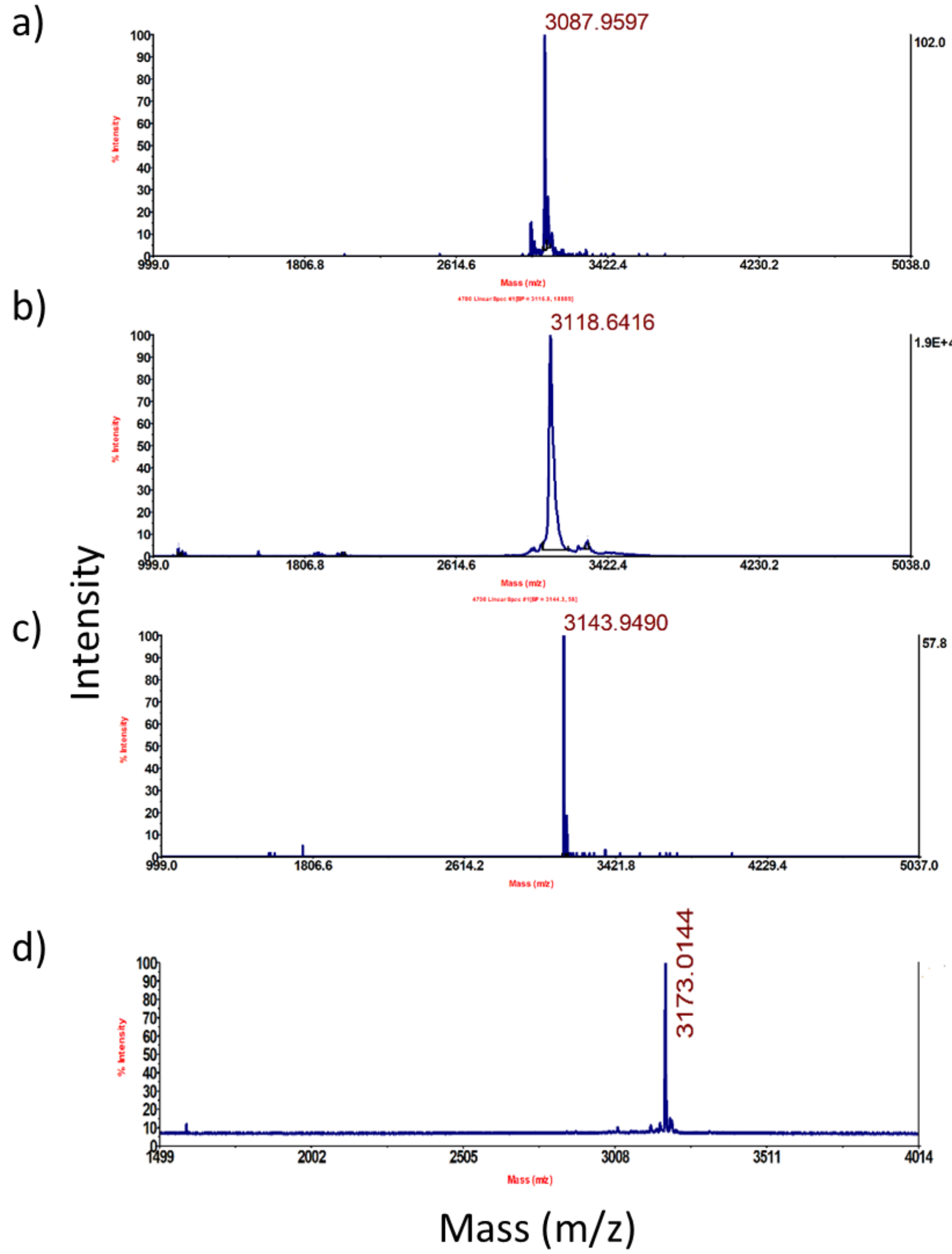


**Fig. S5** MALDI-TOF MS analysis of nisin(1-31) with different lipid chains attached. (a) MS analysis of nisin(1-31)+C4 (compound **6**). (b) MS analysis of nisin(1-31)+C6 (compound **7**). (c) MS analysis of nisin(1-31)+C8 (compound **8**). (d) MS analysis of nisin(1-31)+C10 (compound **9**). The predicted and observed masses are provided in Table S3.

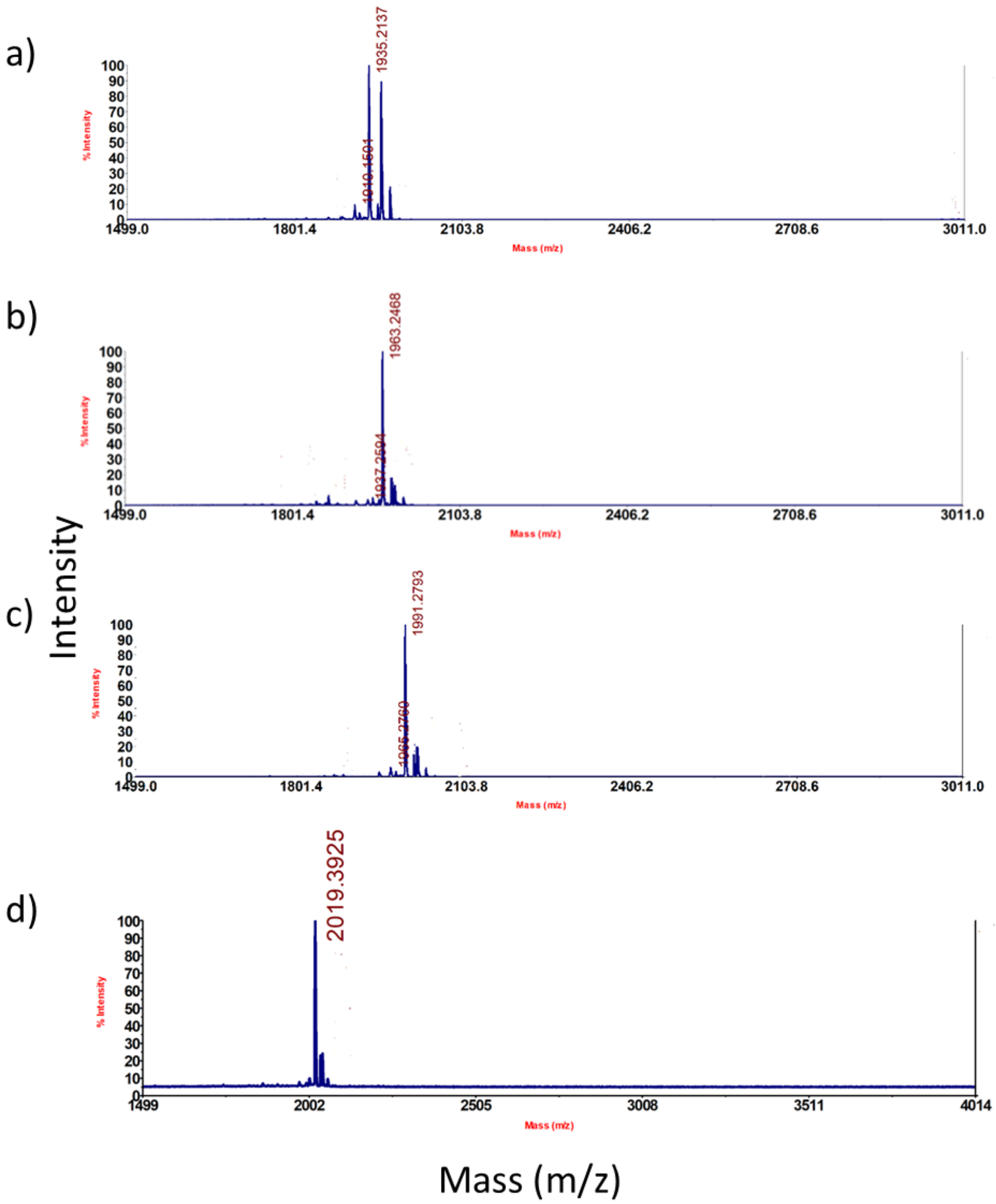


**Fig. S6** MALDI-TOF MS analysis of nisin(1-20) with different lipid chains attached. (a) MS analysis of nisin(1-20)+C4 (compound **10**). (b) MS analysis of nisin(1-20)+C6 (compound **11**). (c) MS analysis of nisin(1-20)+C8 (compound **12**). (d) MS analysis of nisin(1-20)+C10 (compound **13**). The predicted and observed masses are provided in Table S3.

Compound **12**

a)

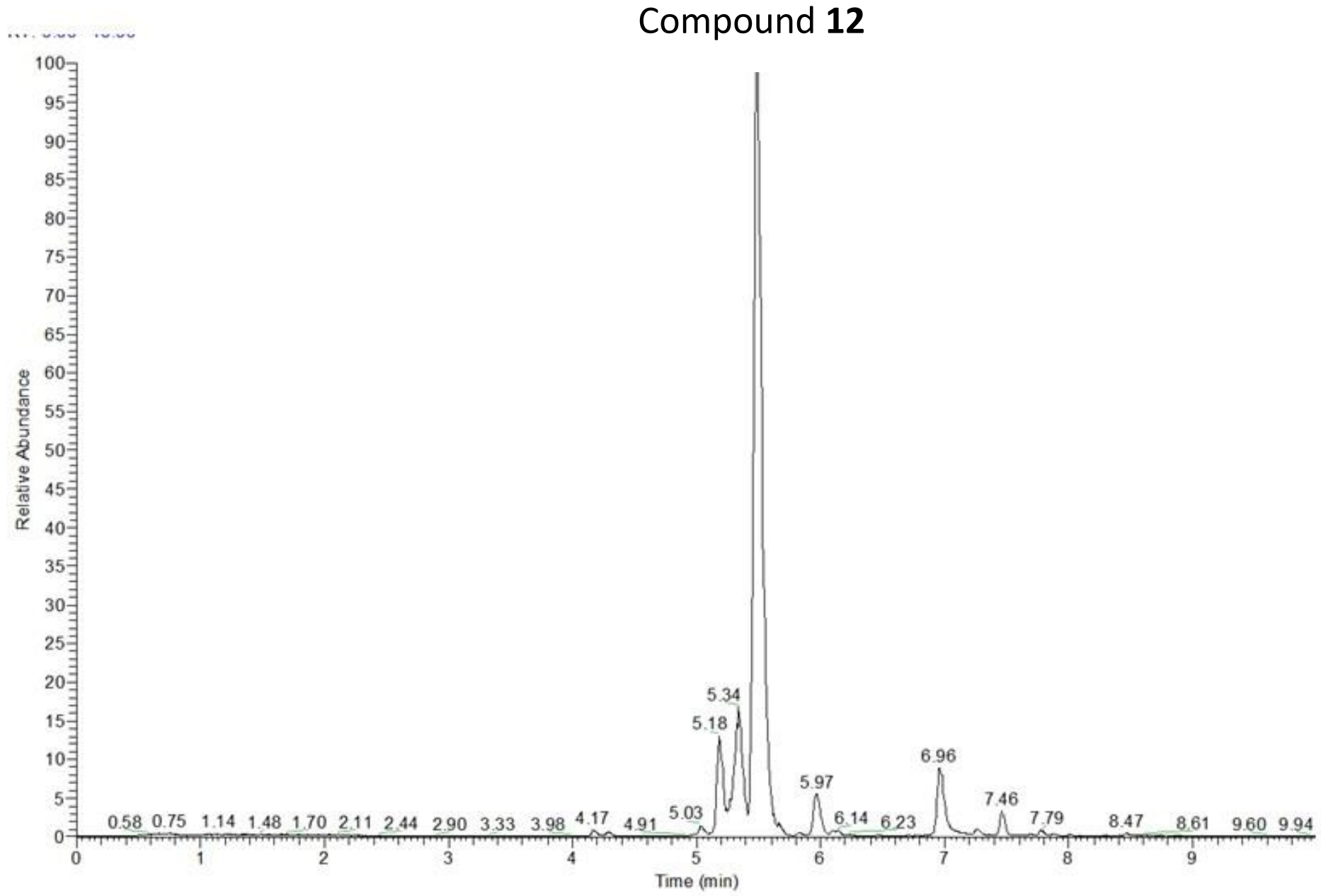


b)

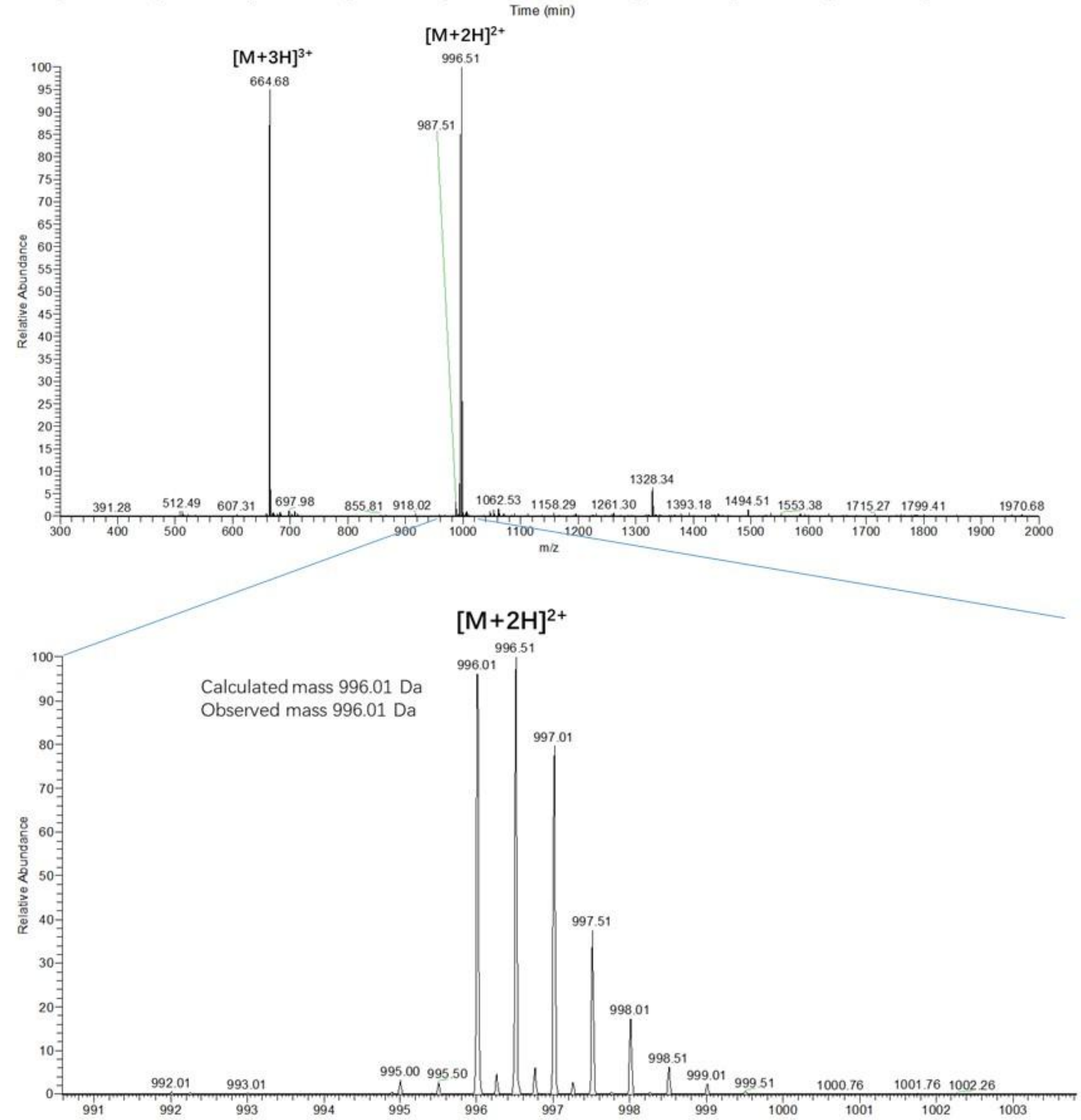

c)

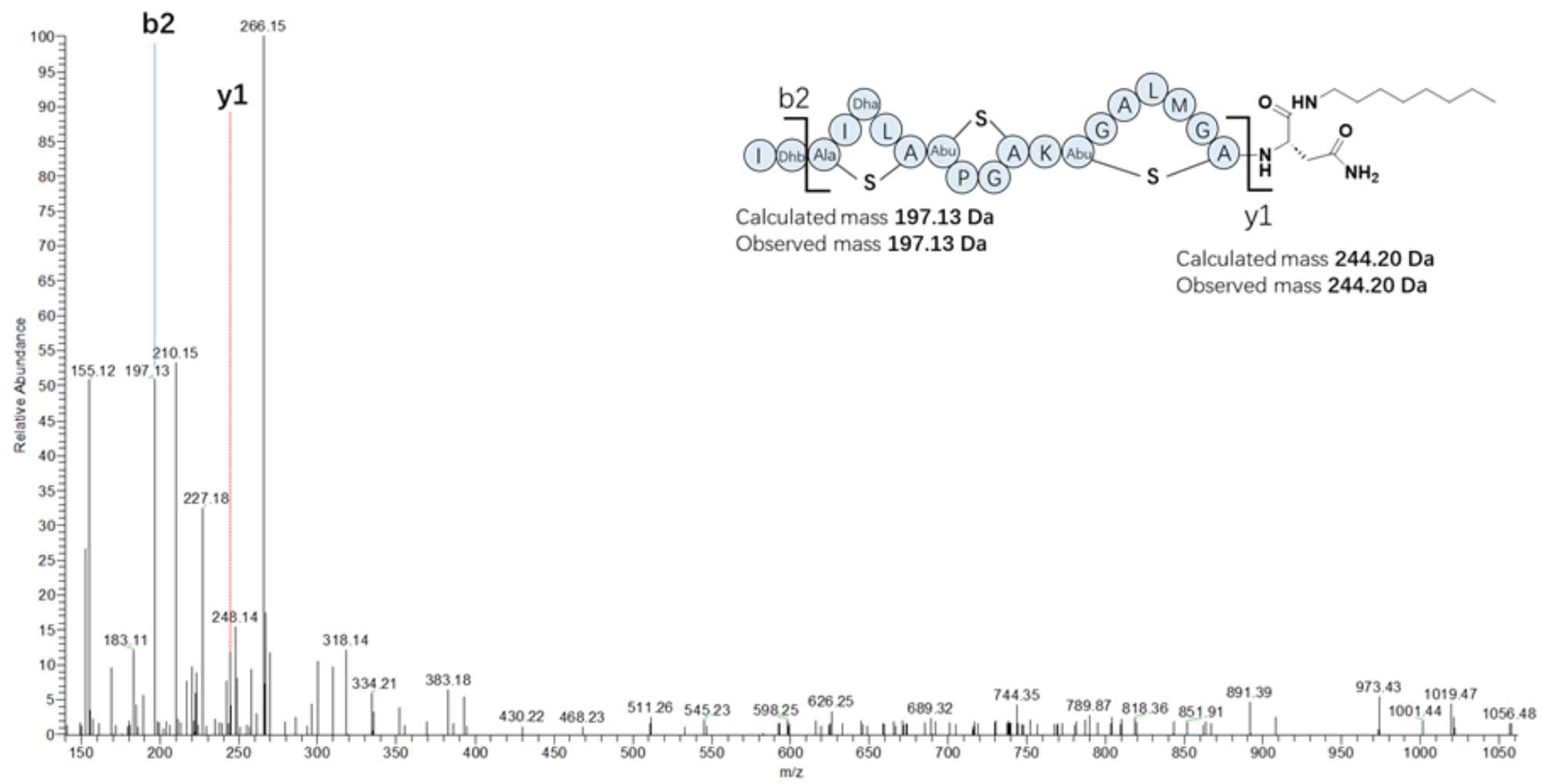


**Fig. S7** High-Resolution Liquid Chromatography-Mass Spectrometry/Mass Spectrometry (HRLC-MS/MS) analysis of HPLC-purified compound **12**. (a) HPLC profile of purified compound **12**. The purity assessment was performed using a Shimadzu LC20 XR-series HPLC system coupled with an Agilent Pursuit XRs $C_8$ column (50 × 2 mm). Detection was carried out at 216 nm. The mobile phase consisted of Eluent A (0.1% formic acid in ultra-pure water) and Eluent B (0.1% formic acid in acetonitrile). The flow rate was set to 0.300 mL/min, with the proportion of Eluent B increasing from 2% to 95% throughout the run, which concluded at 10.0 minutes. (b) High-Resolution MS Spectrum of Compound **12**. The observed mass for the doubly protonated ion $[M+2H]^{2+}$ was 996.01 Da, matching the calculated mass of 996.01 Da, confirming the molecular identity of compound **12**. (c) LC-MS/MS Spectrum of Compound **12**. The spectrum displays characteristic fragment ions, including y1 and b2 ions, contributing to elucidate the compound structure.

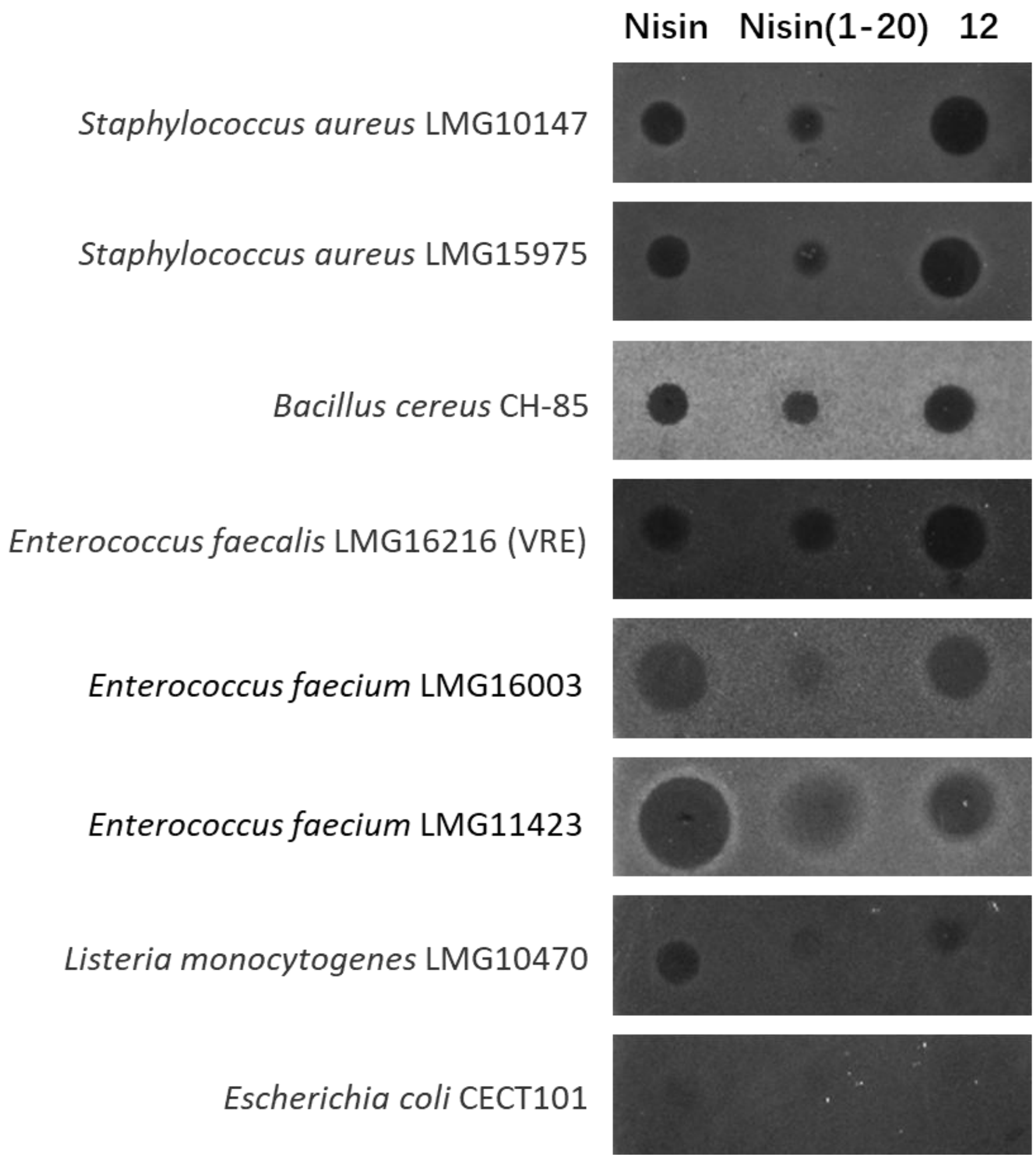


**Fig. S8** A spot-on-lawn assay was used to evaluate the antibacterial spectrum of nisin, nisin(1-20) and compound **12**. A representative image from three independent experiments is shown.

**Table S1** Detailed information about the aliphatic amines used in this study.

| Name | Abbreviation | Company | CAS no. | Molecular Weight |
|---|---|---|---|---|
| Butylamine | C4 | Sigma-Aldrich | 109-73-9 | 73.14 |
| Hexylamine | C6 | Sigma-Aldrich | 111-26-2 | 101.19 |
| Octylamine | C8 | Sigma-Aldrich | 111-86-4 | 129.24 |
| Decylamine | C10 | Sigma-Aldrich | 2016-57-1 | 157.3 |

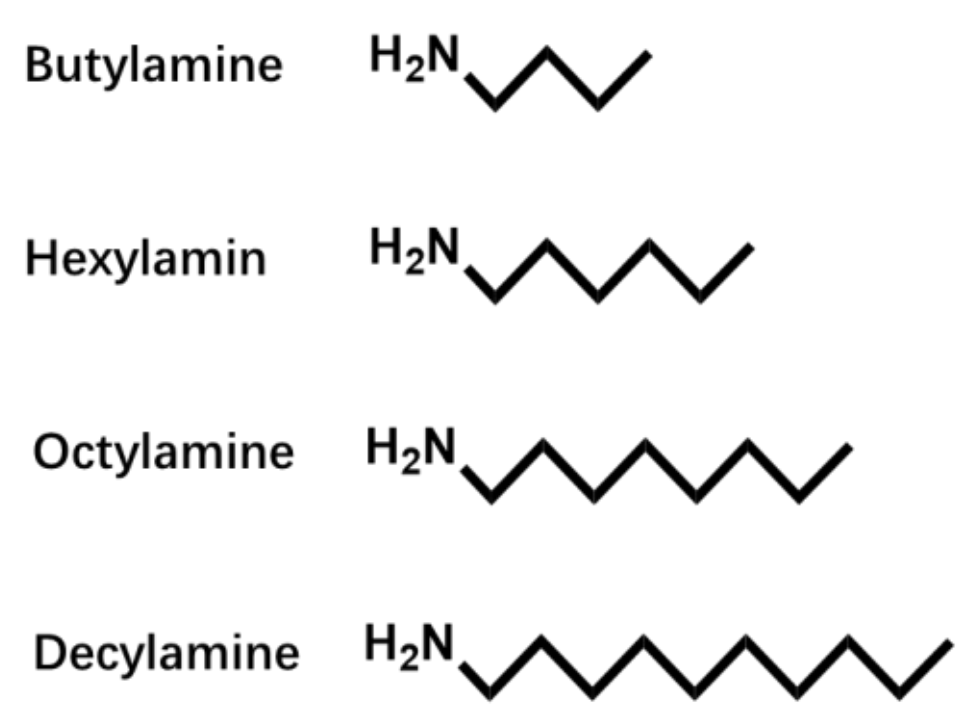

**Table S2** Bacterial strains used in this study.

| Strains | Characteristics | Growth conditions |
|---|---|---|
| *Bacillus cereus* CH-85 | indicator strain | LB medium, 37℃, 180 rpm, 12 h |
| *Lactococcus lactis* MG1363 | indicator strain | GM17 medium, 30℃, 12 h |
| *Staphylococcus aureus* LMG15975 | indicator strain, MRSA | LB medium, 37℃, 180 rpm, 12 h |
| *Staphylococcus aureus* LMG10147 | indicator strain | LB medium, 37℃, 180 rpm, 12 h |
| *Enterococcus faecium* LMG16003 | indicator strain, VRE | GM17 medium, 37℃, 180 rpm, 12 h |
| *Enterococcus faecium* LMG11423 | indicator strain | GM17 medium, 37℃, 180 rpm, 12 h |
| *Enterococcus faecalis* LMG16216 | indicator strain, VRE | GM17 medium, 37℃, 180 rpm, 12 h |
| *Listeria monocytogenes* LMG10470 | indicator strain | BHI medium, 37℃, 180 rpm, 12 h |
| *Escherichia coli* CECT101 | indicator strain | LB medium, 37℃, 180 rpm, 12 h |

MRSA, methicillin-resistant *Staphylococcus aureus*.
VRE, vancomycin-resistant Enterococci.

**Table S3** Comparison of theoretical average masses and MALDI-TOF measured masses of peptides produced in this study.

| Name | Abbreviation | Predicted mass (Da) | Measured mass (Da) | Figure |
|---|---|---|---|---|
| Compound **1** | nisin(-$H_2O$) | 3314.09 | 3314.89 | Fig. 2b |
| nisin(1-31) | | 3033.35 | 3032.91 | Fig. S3a |
| nisin(1-21) | | 2012.52 | 2012.54 | Fig. S3b |
| nisin(1-20) | | 1879.89 | 1879.56 | Fig. S3c |
| nisin(21-31) | | 1172.51 | 1171.53 | Fig. S3d |
| Compound **2** | nisin+C4 | 3387.24 | 3385.84 | Fig. S4a |
| Compound **3** | nisin+C6 | 3415.29 | 3415.67 | Fig. S4b |
| Compound **4** | nisin+C8 | 3443.34 | 3443.55 | Fig. S4c |
| Compound **5** | nisin+C10 | 3471.40 | 3472.64 | Fig. S4d |
| Compound **6** | nisin(1-31)+C4 | 3088.49 | 3087.96 | Fig. S5a |
| Compound **7** | nisin(1-31)+C6 | 3116.54 | 3118.64 | Fig. S5b |
| Compound **8** | nisin(1-31)+C8 | 3144.59 | 3143.95 | Fig. S5c |
| Compound **9** | nisin(1-31)+C10 | 3172.65 | 3173.01 | Fig. S5d |
| Compound **10** | nisin(1-20)+C4 | 1935.03 | 1935.21 | Fig. S6a |
| Compound **11** | nisin(1-20)+C6 | 1963.08 | 1963.25 | Fig. S6b |
| Compound **12** | nisin(1-20)+C8 | 1991.13 | 1991.28 | Fig. S6c |
| Compound **13** | nisin(1-20)+C10 | 2019.19 | 2019.39 | Fig. S6d |
